# Automated decision support tool for static angle modulated ports configuration in VMAT with dynamic collimator rotation for head and neck cancer

**DOI:** 10.1002/acm2.70813

**Published:** 2026-09-23

**Authors:** Hideaki Hirashima, Takahiro Iwai, Shinya Hiraoka, Mitsuhiro Nakamura, Ryota Nakashima, Takashi Mizowaki

**Affiliations:** ^1^ Department of Radiation Oncology and Image‐Applied Therapy, Graduate School of Medicine Kyoto University Kyoto Japan; ^2^ Department of Advanced Medical Physics, Graduate School of Medicine Kyoto University Kyoto Japan

**Keywords:** collimator angle, decision support tool, eclipse scripting application programming interface, RapidArc Dynamic, static angle modulated ports

## Abstract

**Background:**

RapidArc Dynamic (RAD; Varian Medical Systems, Palo Alto, CA) is a novel volumetric modulated arc therapy (VMAT) technique that features dynamic collimator rotation synchronized with gantry rotation. RAD enables the use of static angle‐modulated ports (STAMPs) defined by user selected gantry and collimator angles. However, the determination of STAMP configurations currently relies on manual trial‐and‐error procedures based on planner experience, which limits the efficient clinical implementation of this functionality.

**Purpose:**

This study aimed to develop an automated decision support tool using an Eclipse Scripting Application Programming Interface (ESAPI; Varian Medical Systems) to identify cost‐optimized STAMP configurations and evaluate its clinical utility in complex head and neck cancer planning.

**Methods:**

Twenty patients with nasopharyngeal or sinonasal cancer who had previously received radiotherapy at our institution were retrospectively analyzed. Four treatment plans were generated for each patient: conventional VMAT using RapidArc (RA, Varian Medical Systems), RAD with manually defined STAMPs (RADm) by an expert radiation oncologist, RAD with a vendor‐provided automatic collimator rotation algorithm (RADa), and RAD using the proposed decision support tool (RADdst). The developed algorithm determined the STAMP configurations in three phases: (1) generation of a two‐dimensional geometric cost map across the gantry and collimator angles by integrating planning target volume‐fit and organ‐at‐risk (OAR)‐avoidance terms, (2) extraction of a cost‐minimizing collimator trajectory, and (3) identification of cost‐optimized STAMP positions in geometrically critical regions that require substantial collimator rotation while satisfying machine‐specific rotation‐speed constraints. The dosimetric indices of the targets and OARs, optimization calculation times, and estimated beam delivery times were evaluated.

**Results:**

The ESAPI‐based tool successfully generated dosimetrically feasible RADdst plans for all patients, automatically selecting between two and ten STAMPs according to anatomical complexity. All planning techniques achieved clinically acceptable target coverage and OAR sparing. In nasopharyngeal cancer cases, all RAD approaches significantly reduced the oral cavity dose compared with RA (RADm, *p* = 0.02; RADa, *p* = 0.002; RADdst, *p* = 0.002), and RADdst additionally achieved a significant reduction in the left lens dose (*p* = 0.008). In sinonasal cancer cases, significant differences were observed among the techniques for seven OAR dosimetric metrics (*p* < 0.05), whereas the target coverage remained comparable across all techniques. The optimization calculation time was reduced by approximately 40% for all RAD approaches compared with RA (*p* < 0.01). Furthermore, all RAD techniques reduced the estimated beam delivery time by approximately 20% relative to RA (*p* < 0.01).

**Conclusions:**

An automated ESAPI‐based decision support tool for STAMP configuration in RAD planning was successfully developed. The proposed geometric cost‐based framework achieved a dosimetric performance comparable to both expert‐defined manual STAMP planning and the vendor‐provided automatic collimator rotation approach, while eliminating the need for manual STAMP determination. These findings support the feasibility of the automated STAMP configuration as a practical strategy for enabling an efficient and planner‐independent implementation of RAD planning.

## 1 INTRODUCTION

Volumetric modulated arc therapy (VMAT) has become a standard modality in modern radiotherapy because it provides shorter treatment delivery times and highly conformal dose distributions compared with static‐port intensity‐modulated radiation therapy (IMRT).^1^ VMAT achieves this through the dynamic modulation of gantry rotation speed, multileaf collimator (MLC) leaf positions, and dose rate during treatment delivery. However, the collimator angle is fixed throughout each arc.^2^ Suboptimal collimator angle selection can compromise treatment quality by increasing the dose to organs‐at‐risk (OARs) and limiting target coverage.^3^, ^4^, ^5^, ^6^, ^7^ Particularly, when critical OARs are located adjacent to the planning target volume (PTV), the MLC may excessively shield portions of the target to satisfy OAR dose constraints, thereby reducing the degrees of freedom available for dose modulation and degrading dose homogeneity within the target. Consequently, the collimator angle selection remains an important factor that influences the VMAT plan quality.

RapidArc Dynamic (RAD; Varian Medical Systems, Palo Alto, CA, USA) has recently been introduced as an advanced VMAT optimization technique that incorporates dynamic collimator rotation during gantry rotation.^2^, ^8^, ^9^, ^10^, ^11^, ^12^, ^13^ RAD combines the delivery efficiency of VMAT with the directional modulation capability of static‐port IMRT. A key feature of RAD is its ability to define static angle‐modulated ports (STAMPs), which are characterized by user‐specified gantry and collimator angles. Within the treatment planning system (TPS), STAMPs can be configured either manually, whereby the planner defines all STAMP parameters, or semiautomatically, whereby the planner specifies each gantry angle of the STAMP, and the TPS subsequently determines the remaining collimator rotations during gantry motion. By introducing strategically placed STAMPs, RAD enables enhanced modulation capability at selected gantry angles and has the potential to further improve dosimetric quality.

Despite these advantages, determining appropriate STAMP configurations remains a major challenge in clinical RAD planning.^8^, ^9^, ^10^, ^11^, ^12^, ^13^ Clark et al.^8^ identified the selection of STAMP number, location, and weighting as one of the primary barriers to routine clinical implementation of RAD. Currently, STAMP configuration is commonly determined through planner‐dependent trial‐and‐error processes or predefined institutional templates. Consequently, the full potential of RAD has not been consistently realized in clinical practice.

In this study, we aimed to develop an automated decision support tool that determines geometrically optimized STAMP configurations based on the spatial relationships between the PTV and surrounding OARs. The proposed method evaluates the gantry‐specific geometric characteristics and automatically identifies the STAMP locations that are expected to provide the greatest dosimetric benefits. We evaluated the clinical utility of this approach by comparing the treatment plans generated using the proposed method with those generated using conventional VMAT, manually configured RAD, and the vendor‐provided automatic collimator rotation approach. The effects on planning efficiency, OAR sparing, target dose characteristics, and treatment delivery efficiency were comprehensively assessed.

## 2 METHODS

### 2.1 Patient selection and prescription

This retrospective study was approved by the institutional review board (approval number: 1446‐3).

Twenty patients with head and neck cancer who had previously received radiotherapy at our institution were retrospectively included in this study: 10 with nasopharyngeal cancer and 10 with sinonasal cancer. The target volumes included the gross tumor volume and adjacent high‐risk regions according to the institutional treatment protocols.

For nasopharyngeal cancer, the prescribed dose was 70 Gy in 35 fractions to the primary PTV (PTV70), with dose normalization such that 95% of the target volume received 100% of the prescribed dose (D_95%_ = 70 Gy). A simultaneous integrated boost (SIB) technique was used to deliver 70, 63, and 56 Gy to the PTV70, intermediate‐risk PTV (PTV63), and low‐risk PTV (PTV56), respectively.

For sinonasal cancer, the prescribed dose was 70 Gy in 35 fractions to PTV70, with dose normalization such that 50% of the target volume received 100% of the prescribed dose (D_50%_ = 70 Gy). The SIB technique was used to deliver 70 and 63 Gy to PTV70 and PTV63, respectively.

### 2.2 Treatment planning setting and optimization

The dose constraints for nasopharyngeal and sinonasal cancers are summarized in Table S1. For each patient, four treatment plans were generated and compared: conventional VMAT using RapidArc (RA; Varian Medical Systems), RAD with manually configured STAMPs by an expert radiation oncologist with more than 10 years of clinical experience (RADm), RAD with the vendor‐provided automatic collimator rotation algorithm (RADa), and RAD using the proposed decision support tool (RADdst).

- RA: Conventional VMAT plans consisted of three coplanar arcs with fixed collimator angles of 30°, 330°, and 90°. These angles represent the standard clinical protocol at our institution and were selected to minimize interleaf leakage while maintaining sufficient modulation capability.
- RADm: RAD plans consisted of two coplanar arcs. The STAMP configurations, including the number of STAMPs and their corresponding gantry and collimator angles, were manually determined by an experienced radiation oncologist. Specifically, four STAMPs per arc were placed at fixed gantry angles: 210°, 290°, 70°, and 150° for Arc 1 and 240°, 320°, 40°, and 120° for Arc 2. Continuous collimator rotation between adjacent STAMPs was optimized using the RAD optimization algorithm.
- RADa: RAD plans consisted of two coplanar arcs. For each arc, a single initial STAMP was manually placed at the starting control point at a fixed gantry angle consistently across all patients: 160° for Arc 1 and 190° for Arc 2. No additional STAMPs were specified. The Eclipse RAD optimization algorithm (version 18.1) was subsequently used to determine the remaining continuous collimator rotations throughout the gantry motion.
- RADdst: RAD plans consisted of two coplanar arcs. STAMP configurations, including the number of STAMPs and their corresponding gantry and collimator angles, were determined using the proposed Eclipse Scripting Application Programming Interface (ESAPI; Varian Medical Systems)‐based decision support tool. The generated STAMP parameters were subsequently transferred to the TPS for plan optimization. The details of the algorithm are described in Section 2.3.

All treatment plans were optimized using a Photon Optimizer, and dose distributions were calculated using Acuros XB with a dose calculation grid size of 2.0 mm. For RAD planning, the STAMP weight parameters were set to −1 (Arc mode) for nasopharyngeal cancer and −2 (Arc‐dominant mode) for sinonasal cancer, according to institutional planning practice. The maximum number of optimization iterations for all the RAD plans was set to 2000.

The optimization objectives were first established using the RA plan to ensure a fair comparison among the planning techniques. The RA plan was optimized up to three times until the acceptable dose constraints were met. The resulting objective template was subsequently applied to the corresponding RAD plans. If the predefined planning goals were not satisfied, an additional optimization cycle was permitted.

### 2.3 Automated decision support tool for RAD

A custom decision support tool was developed using ESAPI v18.1 to reduce the planner‐dependent trial‐and‐error process associated with RAD planning. The source code of the ESAPI script is openly available on GitHub (https://github.com/HideakiHirashima/ESAPI‐RAD‐STAMP‐Optimizer). The proposed algorithm evaluates the geometric relationships between the PTV and surrounding OARs in the beam's‐eye view (BEV), while incorporating the mechanical constraints of TrueBeam with Millennium 120 MLC (Varian Medical Systems, Palo Alto, CA). Based on these factors, the algorithm automatically determines the geometrically optimized STAMP configurations, including the number of STAMPs, their locations (gantry angles), and the corresponding collimator angles.

**FIGURE 1:**
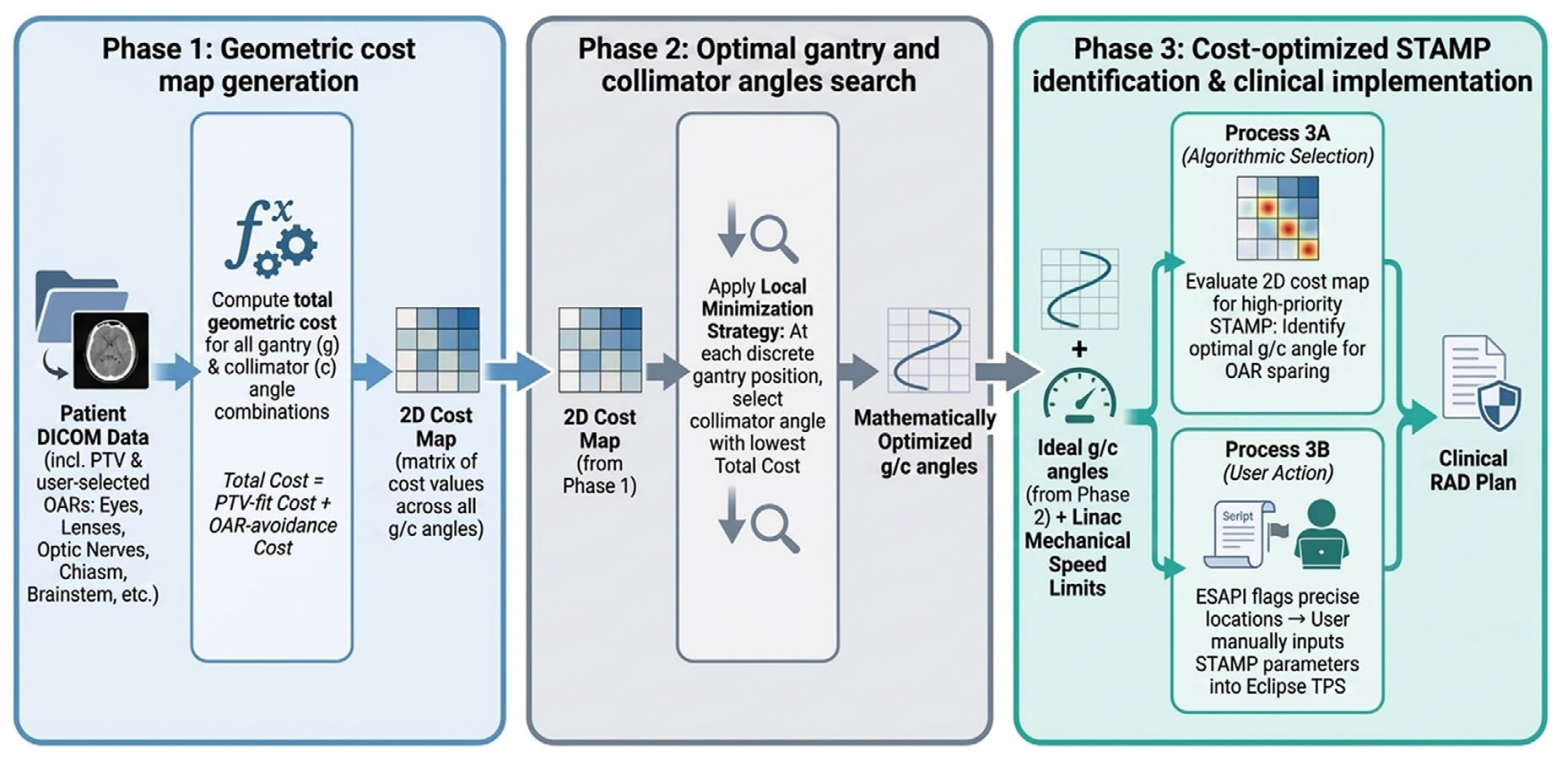
Workflow of decision support tool for STAMP configuration. Abbreviations: ESAPI, eclipse scripting application programming interface; OAR, organ‐at‐risk; PTV, planning target volume; RAD, RapidArc dynamic; STAMP, static angle‐modulated port; TPS, treatment planning system; 2D, two‐dimensional.

As illustrated in Figure 1, the workflow consists of three sequential phases: (1) geometric cost map generation, (2) collimator trajectory optimization, and (3) STAMP identification. These steps enable the automatic derivation of dosimetrically feasible STAMP configurations that balance target coverage, OAR avoidance, and delivery efficiency.

#### 2.3.1 Geometric cost map generation

For a complete gantry rotation, the algorithm first extracts the contours of the PTV and user‐selected OARs. A composite geometric cost is subsequently calculated for every combination of gantry angle (g) and collimator angle (*c*). The resulting two‐dimensional (2D) cost map serves as the basis for subsequent collimator trajectory optimization and STAMP identification.

The composite cost consists of two components: a PTV‐fit term and an OAR‐avoidance term. The PTV‐fit term evaluates the efficiency with which the MLC conforms to the projected target shape in the BEV. As illustrated in Figure 1 when the collimator is aligned with the major axis of the projected PTV, the required MLC aperture bounding box is minimized. This generally reduces unnecessary aperture expansion and increases the degrees of freedom available for beam modulation. Consequently, the collimator orientations that produce a smaller projected bounding box are assigned lower PTV‐fit costs. The PTV‐fit cost is defined as follows:


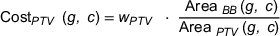

where $\;\mathrm{Are}a_{\;BB}\left(g,\;c\right)$ denotes the area of the minimum bounding box enclosing the projected PTV at gantry angle (g) and collimator angle (*c*), 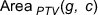 denotes the projected PTV area in the BEV, and $w_{PTV}$ is a weighting coefficient controlling the contribution of the target‐conformity term. Lower values of $\mathrm{Cos}t_{PTV}\left(g,\;c\right)$ indicate a more efficient geometric alignment between the projected target and MLC aperture.

The OAR‐avoidance term evaluates the geometric relationship between the PTV and user‐selected OARs. This term penalizes the collimator orientations in which the MLC leaf travel direction simultaneously intersects both the PTV and adjacent OARs. Such configurations may increase the difficulty of achieving adequate target coverage while maintaining OAR sparing. In contrast, when the MLC leaf motion direction is aligned parallel to the interface between the PTV and OAR, the target and OAR can be more effectively separated by the leaf motion, potentially reducing dose spillage into normal tissues. The OAR‐avoidance cost is calculated as follows:


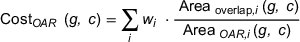

where $\;\mathrm{Are}a_{\;\mathrm{overlap},i}\left(g,\;c\right)$ denotes the overlap area between the projected PTV and the *i*‐th OAR in the BEV, evaluated with respect to the MLC leaf travel direction, 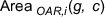 denotes the projected area of the *i*‐th OAR, and $w_{i}$ is an OAR‐specific weighting factor reflecting its dosimetric evaluation. Consequently, larger overlap ratios result in higher OAR‐avoidance costs, whereas collimator orientations that facilitate the independent modulation of the PTV and OAR incur lower costs. In this study, the user‐selected OARs included the eyes, lenses, optic nerves, optic chiasm, brainstem, larynx, spinal cord, cochleae, oral cavity, and parotid glands. The total geometric cost for a given gantry angle (g) and collimator angle (*c*) was calculated as the sum of the PTV‐fit and OAR‐avoidance terms:

$$\mathrm{Cos}t_{\mathrm{total}}\;\left(g,\;c\right)=\;\mathrm{Cos}t_{PTV}\left(g,\;c\right)+\;\mathrm{Cos}t_{OAR}\left(g,\;c\right)$$

Lower values of $\mathrm{Cos}t_{\mathrm{total}}\left(g,\;c\right)$ indicate collimator orientations that provide a more favorable geometric balance between the target conformity and OAR avoidance. By evaluating $\mathrm{Cos}t_{\mathrm{total}}\left(g,\;c\right)$ for all combinations of gantry and collimator angles, a 2D geometric cost map was generated. This cost map represents the geometric suitability of each gantry–collimator configuration and serves as the basis for the subsequent collimator trajectory optimization and STAMP identification.

#### 2.3.2 Collimator trajectory optimization

After generating the 2D cost map, the algorithm determines a cost‐minimizing collimator trajectory across the full gantry rotation. For each discrete gantry angle (g), all the candidate collimator angles (*c*) are evaluated, and the collimator angle associated with the minimum total geometric cost is selected as follows:

$$c^{*}\;\left(g\right)=\arg\min_{c}\mathrm{Cos}t_{\mathrm{total}}\left(g,\;c\right)$$

where $c^{*}\left(g\right)$ represents the cost‐minimizing collimator angle at gantry angle (g). Repeating this process for all the gantry positions yields a continuous raw collimator trajectory. The minimum geometric cost associated with this optimized angle at each gantry position is extracted as the geometric optimality profile, which is defined as follows:

$$\mathrm{Scor}e_{\mathrm{geom}}\;\left(g\right)=\;\mathrm{Cos}t_{\mathrm{total}}\left(g,\;c^{*}\left(g\right)\right)$$

This trajectory and its corresponding cost profile balance the target conformity with OAR avoidance, based on the composite geometric cost function.

#### 2.3.3 Static angle modulated port identification based on cost evaluation and mechanical constraints

Although the raw cost‐minimizing collimator trajectory provides the geometrically preferred collimator orientation at each gantry angle, the direct delivery of this trajectory may not be clinically feasible because the required collimator rotation speed can exceed the mechanical limits of TrueBeam. Therefore, an additional step was implemented to identify strategically placed STAMPs by evaluating a comprehensive score that balances the mechanical delivery difficulty with the previously defined geometric optimality. The algorithm identifies optimal STAMP locations through a multistep numerical optimization process. First, a mechanical constraint term, $\mathrm{Scor}e_{\mathrm{mech}}\left(g\right)$, is calculated at each gantry angle (g) to evaluate the required collimator rotational velocity relative to the machine limits:

$$\mathrm{Scor}e_{\mathrm{mech}}\left(g\right)=\left|\frac{c^{*}\left(g_{i}\right)-c^{*}\left(g_{i-1}\right)}{\Delta g}\right|\;\cdot \frac{\omega_{\mathrm{gantry}}}{\omega_{\mathrm{coll}}}$$

where $c^{*}\left(g_{i}\right)-$ $c^{*}\left(g_{i-1}\right)$ represents the change in the cost‐minimizing collimator angle between adjacent discrete gantry positions, Δg is the gantry angle interval, and $\omega_{\mathrm{gantry}}$ and $\omega_{\mathrm{coll}}$ represent the maximum mechanical rotation speeds of the gantry and collimator, respectively. In this study, these machine limits were set to $\omega_{\mathrm{gantry}}=6.0^{\circ }/s$ and $\omega_{\mathrm{coll}}=9.0^{\circ }/s$, which correspond to the vendor‐recommended standard specifications for TrueBeam. Second, a comprehensive selection metric, $\mathrm{Scor}e_{\mathrm{STAMP}}\left(g\right)$, is established by combining the normalized geometric cost profile, $\mathrm{Scor}e_{\mathrm{geom}}\left(g\right)$, and the mechanical constraint term using a weighted linear combination:

$$\mathrm{Scor}e_{\mathrm{STAMP}}\;\left(g\right)=\;\mathrm{Scor}e_{\mathrm{geom}}\left(g\right)+\;\mathrm{Scor}e_{\mathrm{mech}}\left(g\right)$$

High $\mathrm{Scor}e_{\mathrm{STAMP}}\left(g\right)$ values effectively pinpoint critical gantry regions where rapid collimator rotation is geometrically essential to spare OARs but is mechanically restricted, thereby necessitating a STAMP. Third, candidate STAMP locations are determined by identifying the local maxima of the $\mathrm{Scor}e_{\mathrm{STAMP}}\left(g\right)$ distribution across the full arc. To prevent over‐clustering of STAMP and ensure that it is dosimetrically feasible, a proximity‐filter constraint is applied; if multiple candidate peaks are identified within a predefined angular threshold ($\Delta \;\theta_{\mathrm{threshold}}=20^{\circ }$), a local spacing clearance is enforced, prioritizing the candidate locations with the highest composite scores. By establishing STAMPs at these mathematically prioritized locations, the algorithm preserves highly favorable low‐cost collimator orientations, while ensuring that the final trajectory remains machine‐specific mechanical constraints within the machine limits of TrueBeam.

### 2.4 Plan evaluation metrics

Plan quality was evaluated in terms of dosimetric performance, operational efficiency, and delivery complexity.

For dosimetric performance, the target coverage was assessed using the dose received by 95% of the PTV (D_95%_) for all PTVs. OAR sparing was evaluated by recording the maximum dose (D_max_) to the lenses, eyes, optic nerves, optic chiasm, temporal lobes, and brainstem and the mean dose (D_mean_) to the cochleae, larynx, oral cavity, and bilateral parotid glands.

The operational efficiency was assessed using the total optimization calculation time and the estimated beam delivery time. The optimization calculation time was defined as the total wall‐clock computation time required for the planning process, measured from the initiation of the optimization algorithm in Eclipse TPS (v18.1) through the completion of the final dose calculation step (optimization and Acuros XB calculation). The estimated beam delivery time was automatically evaluated using a custom script that parsed the plan control‐point details (https://github.com/Varian‐MedicalAffairsAppliedSolutions/PlanDeliverySimulator). The script calculated the total delivery duration by evaluating gantry movement speed, collimator rotation speed, and MU delivery rates across consecutive control points in accordance with the TrueBeam hardware mechanical kinematic limits.

Delivery complexity was quantified using the total number of monitor unit (MU) and the modulation complexity score (MCS),^14^ which characterizes the variations in MLC leaf positions and aperture shapes throughout treatment delivery. Lower MCS values indicate a greater degree of modulation and increased plan complexity.

### 2.5 Statistical analyses

Statistical analyses were performed to compare the dosimetric and treatment efficiency metrics of the four planning techniques (RA, RADm, RADa, and RADdst) for each treatment site.

The overall differences among the four planning techniques were assessed using the Friedman test. When a significant difference was detected, pairwise comparisons were performed using the Wilcoxon signed‐rank test. Holm correction was applied as a control for multiple comparisons. Statistical significance was defined as a two‐sided p‐value of < 0.05, after correction. All statistical analyses were performed using Python version 3.10.

## 3 RESULTS

### 3.1 Performance of the automated decision support tool

The developed ESAPI‐based decision support tool was successfully applied to all cases and generated RAD plans that satisfied the mechanical constraints of TrueBeam. Depending on the geometric complexity of the patient's anatomy, the median number of automatically identified STAMPs was 4 (range, 2–10). The patient‐specific STAMP configurations determined by RADdst across all 20 patients are comprehensively reported in Table S2. The proposed RADdst tool accomplished fully automated, patient‐customized STAMP determination in only 2–3 min without manual trial and error.

**FIGURE 2:**
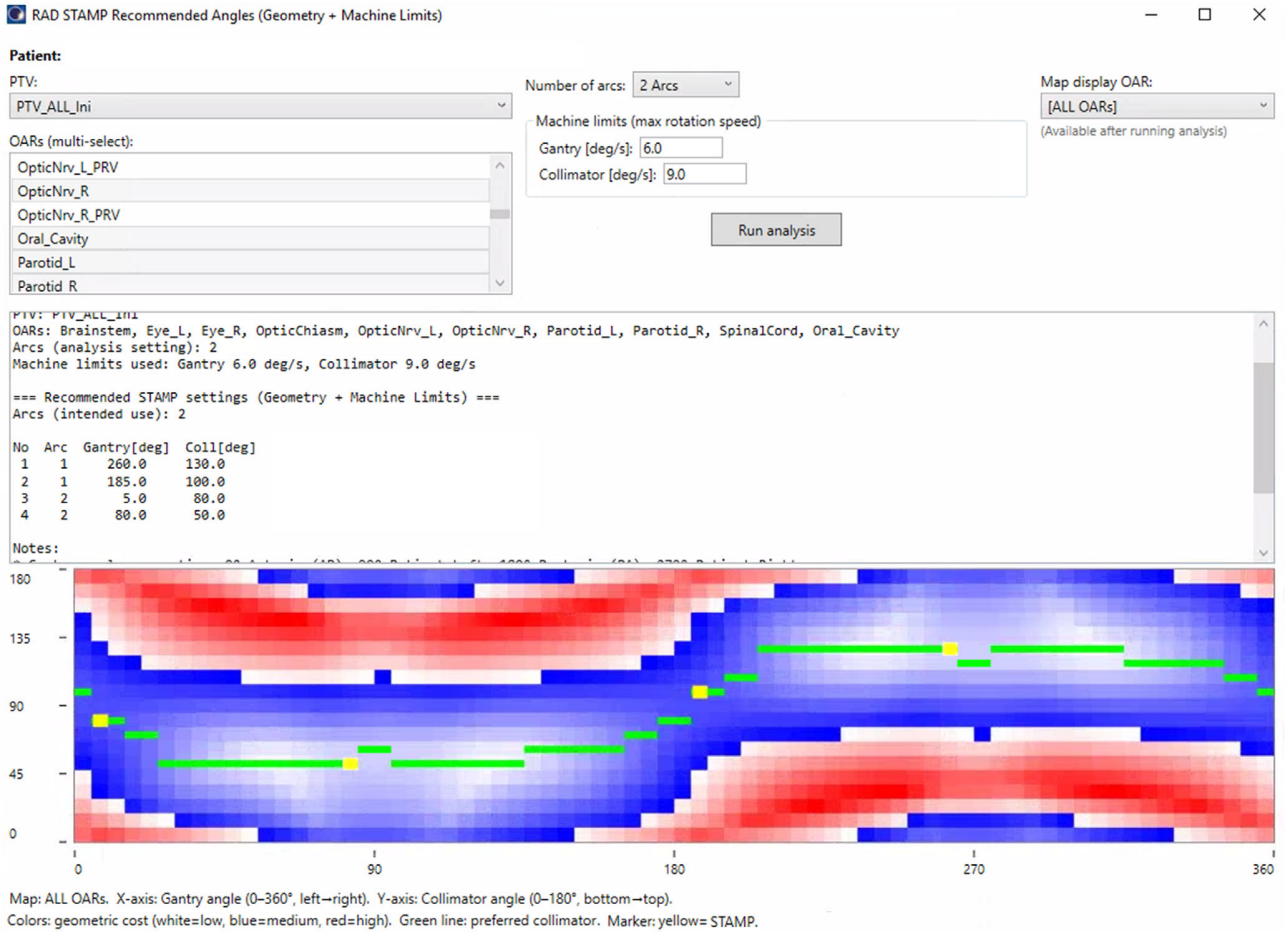
Eclipse scripting application programming interface showing automated STAMP determination. The 2D geometric cost map plots gantry (horizontal axis) and collimator (vertical axis) angles, indicating low‐cost regions (white), medium‐cost regions (blue), high‐cost regions (red), the cost‐minimizing trajectory (green), and identified STAMPs (yellow). Abbreviations: OAR, organ‐at‐risk; PTV, planning target volume; RAD, RapidArc Dynamic; ROI, region of interest; STAMP, static angle‐modulated port.

A representative example of the algorithm output is shown in Figure 2. The generated 2D geometric cost map identified regions of low geometric cost across the gantry–collimator angle space. Based on this cost landscape, the algorithm derived a continuous cost‐minimizing collimator trajectory (green line). Regions in which the required collimator rotation exceeded the maximum allowable rotation speed relative to the gantry motion were automatically identified. These locations were subsequently designated as STAMPs (yellow markers), resulting in a collimator trajectory compliant with the machine‐specific kinematic constraints.

No mechanical constraint violations were observed in any RADdst plan generated using the proposed workflow.

### 3.2 Dosimetric evaluation

**FIGURE 3:**
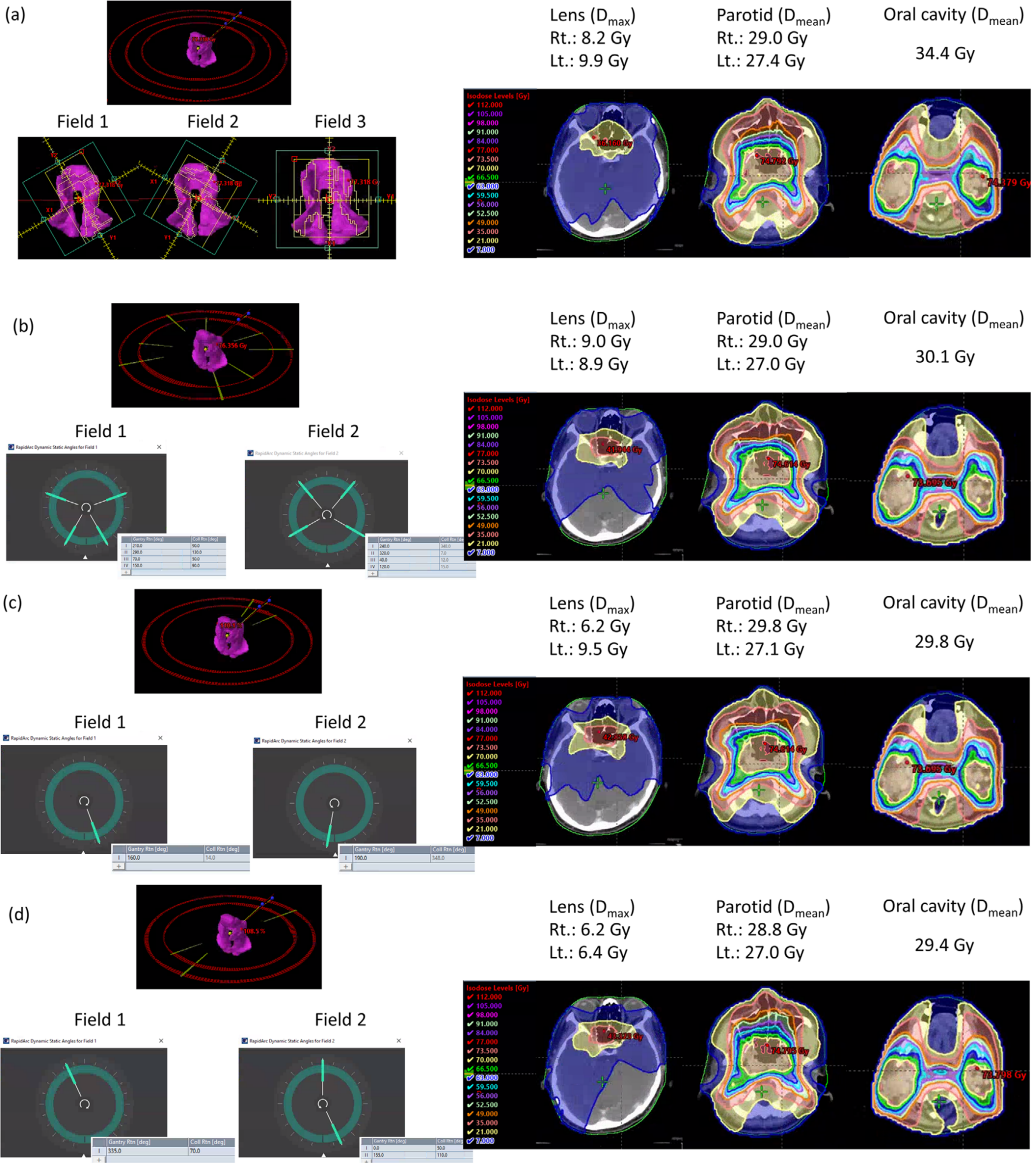
The representative STAMP position and dose distribution in nasopharyngeal cancer for (a) RA, (b) RADm, (c) RADa, and (d) RADdst. Abbreviations: STAMP, static angle‐modulated ports; RA, RapidArc; RAD, RapidArc Dynamic; D_max_, maximum dose to the structure volume; D_mean_, mean dose to the structure volume.

The representative STAMP positions and dose distributions for RA, RADm, RADa, and RADdst are shown in Figure 3, and the quantitative dosimetric results are summarized in Table 1. All four planning techniques achieved clinically acceptable target coverage and OAR sparing. Although statistically significant differences were observed in PTV63 and PTV56 for nasopharyngeal cancer and in CTV70 for sinonasal cancer, the absolute differences were small, and the target coverage was nearly equivalent among all techniques.

**TABLE 1:** Median values (interquartile ranges) of plan evaluation metrics.

		Nasopharyngeal cancer					Sinonasal cancer				
Structure	Index	RA	RADm	RADa	RADdst	Overall *p* value	RA	RADm	RADa	RADdst	Overall *p* value
PTV63	D_95%_ [Gy]	55.5 [54.1–55.7]	54.6 [53.4–55.0]	54.6 [53.9–55.5]	55.4^*^ [54.1–55.5]	0.03	57.5 [57.3–58.4]	57.2 [56.7–59.0]	57.9 [57.6–59.0]	57.0 [56.5–58.0]	0.39
PTV56	D_95%_ [Gy]	52.9 [52.8–53.2]	52.7 [52.5–53.3]	52.5^*^ [52.4–53.2]	52.6^*^ [52.2–53.0]	< 0.01	–	–	–	–	
GTV	D_98%_ [Gy]	70.5 [70.4–70.8]	70.8 [70.6–70.9]	70.5 [70.5–70.8]	70.7 [70.6–70.8]	0.13	61.2 [59.0–64.0]	58.6 [58.3–63.5]	60.1 [57.1–62.7]	58.4 [57.3–63.0]	0.61
CTV70	D_95%_ [Gy]	70.9 [70.9–71.0]	70.9 [70.8–71.0]	70.8 [70.7–70.9]	70.8 [70.8–70.9]	0.06	64.9 [63.0–67.9]	66.7 [65.6–68.1]	66.9^*^ [66.1–68.7]	65.9 [64.8–68.1]	< 0.01
CTV63	D_95%_ [Gy]	60.8 [60.5–60.9]	60.8 [60.4–61.0]	60.9 [60.3–61.1]	61.2 [60.8–61.4]	0.31	62.6 [62.0–63.1]	62.5 [62.2–63.0]	63.1 [62.8–63.5]	62.8 [62.3–63.2]	0.06
CTV56	D_95%_ [Gy]	55.5 [55.4–55.6]	55.6 [55.3–55.8]	55.4 [55.3–55.6]	55.5 [55.4–55.7]	0.26	–	–	–	–	
Spinal cord	D_max_ [Gy]	34.4 [32.2–37.1]	37.2 [36.1–40.7]	32.2 [31.5–36.3]	31.1 [30.7–36.6]	< 0.01	25.6 [24.9–30.4]	29.3 [27.7–31.8]	32.9 [30.7–36.2]	31.5 [31.2–35.1]	0.15
Brainstem	D_max_ [Gy]	55.6 [54.4–57.8]	54.2 [48.2–54.2]	54.3 [49.6–54.3]	52.0 [50.1–55.0]	0.05	42.1 [39.6–46.5]	39.6 [39.2–45.2]	41.7 [39.0–45.4]	42.9 [39.0–45.0]	0.16
Lt eye	D_max_ [Gy]	22.3 [20.6–24.3]	22.0 [19.3–24.8]	22.2 [20.4–23.8]	23.0 [19.3–23.8]	0.47	42.3 [40.5–45.6]	39.1^*^ [36.0–41.5]	38.4^*^ [35.1–41.4]	39.1^*^ [37.0–42.5]	< 0.01
Rt eye	D_max_ [Gy]	20.7 [19.6–21.8]	22.8 [21.7–24.1]	21.9 [17.6–22.9]	22.9 [18.6–23.6]	0.21	42.8 [42.2–46.8]	40.9^*^ [40.0–42.5]	40.5^*^ [39.7–42.2]	40.6^*^ [39.6–42.5]	0.01
Lt lens	D_max_ [Gy]	7.9 [7.3–8.9]	7.8 [5.3–8.8]	6.7 [6.4–8.2]	6.0^*^ [5.9–6.6]	0.04	9.6 [8.8–10.3]	8.1^*^ [7.8–8.8]	7.6^*^ [7.0–7.8]	7.8^*^ [7.4–8.4]	< 0.01
Rt lens	D_max_ [Gy]	6.6 [5.4–7.9]	8.9 [8.5–9.1]	6.0 [5.9–6.2]	6.3 [5.8–8.0]	0.07	8.7 [8.0–11.1]	8.5^*^ [8.2–9.0]	7.9^*^ [7.6–8.6]	8.3^*^ [7.4–9.3]	< 0.01
Lt optic nerve	D_max_ [Gy]	27.3 [22.0–35.2]	36.1 [31.0–44.0]	33.5 [29.5–43.8]	34.8 [26.8–41.9]	0.05	54.0 [53.2–54.4]	53.3 [52.1–54.1]	53.3 [52.2–53.9]	52.9 [52.7–54.3]	0.43
Rt optic nerve	D_max_ [Gy]	28.2 [25.2–29.3]	36.1 [29.4–38.2]	32.7 [31.1–34.1]	33.3 [31.3–35.1]	0.05	53.7 [52.7‐54.3]	53.2 [52.3‐54.5]	52.9 [52.5‐54.0]	53.2 [52.1‐54.5]	0.20
Chiasm	D_max_ [Gy]	14.7 [12.7–18.2]	21.0^*^ [18.7–29.1]	21.4^*^ [15.4–24.9]	16.5^*^ [15.5–26.9]	< 0.01	52.2 [51.4–53.0]	51.4 [50.2–51.8]	51.5 [50.9–51.7]	51.1 [50.3–51.6]	0.14
Lt parotid	D_mean_ [Gy]	28.7 [27.4–32.6]	28.0 [26.9–32.3]	27.5 [26.9–31.4]	27.9 [26.7–31.4]	0.06	23.2 [20.8–27.4]	20.2^*^ [18.0–25.5]	18.7^*^ [17.2–24.3]	19.6^*^ [17.5–24.9]	< 0.01
Rt parotid	D_mean_ [Gy]	28.2 [27.5–28.8]	28.6 [27.4–29.4]	28.0 [26.6–28.6]	28.0 [26.8–28.5]	0.28	28.9 [26.6–32.8]	25.7^*^ [24.1–28.4]	23.3^*^ [21.9–29.0]	24.6^*^ [22.8–28.3]	0.01
Lt cochlea	D_mean_ [Gy]	40.6 [39.8–41.1]	40.8 [40.1–43.4]	40.3 [39.5–41.7]	40.6 [39.9–44.0]	0.06	35.9 [33.8–38.8]	39.0 [33.9–44.0]	40.8 [37.7–43.2]	37.0 [36.6–40.5]	0.32
Rt cochlea	D_mean_ [Gy]	40.6 [40.0–40.9]	40.6 [40.0–41.9]	40.0 [39.8–40.8]	40.2 [39.9–40.7]	0.06	38.4 [32.4–43.4]	40.8 [36.3–45.9]	37.9 [37.5–43.7]	39.9 [37.3–42.1]	0.61
Temporal lobe	D_max_ [Gy]	70.4 [68.3–71.3]	70.9 [69.7–71.1]	70.4 [68.8–70.9]	70.3 [68.4–71.4]	0.24	74.0 [72.7–74.0]	73.6 [72.9–74.0]	73.4 [72.8–73.9]	73.9 [72.7–74.6]	0.43
Larynx	D_mean_ [Gy]	27.6 [26.0–28.9]	24.6^*^ [22.9–25.8]	26.0 [23.7–27.4]	26.0 [24.2–26.8]	< 0.01	33.0 [32.4–42.2]	31.6 [29.5–41.7]	30.1 [27.7–38.9]	34.4 [29.6–44.1]	0.12
Oral cavity	D_max_ [Gy]	40.4 [33.3–45.3]	39.4^*^ [31.7–41.7]	39.1^*^ [28.5–40.6]	38.5^*^ [29.4–39.4]	< 0.01	32.1 [30.5–37.8]	27.0^*^ [26.2–32.8]	28.1^*^ [27.0–34.1]	27.3^*^ [26.2–33.5]	< 0.01
Body	D_max_ [Gy]	77.0 [76.0–77.7]	76.8 [76.4–78.0]	77.2 [77.1–77.7]	76.9 [75.7–77.9]	0.44	77.3 [76.2–79.2]	77.4 [75.9–77.5]	76.2 [75.2–76.5]	77.5 [75.9–77.6]	0.65
Optimize time [s]		242 [236–259]	144^*^ [138–154]	143^*^ [131–156]	140^*^ [128–156]	< 0.01	241 [222–277]	112^*^ [104–120]	104^*^ [100–112]	106^*^ [99–115]	< 0.01
Estimated delivery time [s]		202 [202–202]	172^*^ [171–173]	158^*^ [150–167]	152^*^ [152–154]	< 0.01	196 [196–196]	145^*^ [142–149]	141^*^ [140–141]	145^*^ [138–149]	< 0.01
MU		497 [481–508]	796^*^ [749–820]	665^*^ [647–679]	677^*^ [671–699]	< 0.01	400 [397–475]	710 [666–777]	606 [576–621]	734 [677–737]	< 0.01
MCS		0.34 [0.33–0.35]	0.27^*^ [0.26–0.28]	0.22^*^ [0.21–0.22]	0.20^*^ [0.20–0.20]	< 0.01	0.36 [0.35–0.38]	0.26^*^ [0.25–0.28]	0.21^*^ [0.21–0.22]	0.28^*^ [0.27–0.28]	< 0.01

*Note*: Overall *p* values were calculated using the Friedman test.

Abbreviations: CTV, clinical target volume; Dmax, maximum dose to the structure volume; Dmean, mean dose to the structure volume; Dxx%, maximum dose covering ≥ xx% of the structure volume; MCS, modulation complexity score; MU, monitor unit; PTV, planning target volume;RA, RapidArc; RADa, RAD with automated collimator rotation; RADdst, RAD with automated decision support tool; RADm, RapidArc dynamic with manual static angle modulated ports (STAMPs).

* A statistically significant difference compared with the conventional RA plan (*p* < 0.05, Wilcoxon signed‐rank test with Holm correction). Post‐hoc pairwise comparisons among the three RAD modalities (RADm, RADa, RADdst) revealed no statistically significant differences in these metrics.

For nasopharyngeal cancer, significant overall differences were observed in the spinal cord, left lens, optic chiasm, larynx, and oral cavity according to the Friedman test (*p* = 0.04). Pairwise comparisons demonstrated that all three RAD techniques significantly reduced the D_mean_ to the oral cavity compared with RA (RADm, *p* = 0.02; RADa, *p* = 0.002; RADdst, *p* = 0.002). In addition, the RADdst achieved a significantly lower D_max_ in the left lens than the RA (*p* = 0.008), whereas the RADm yielded a significantly lower laryngeal dose than the RA (*p* = 0.004). A significant difference in the spinal cord dose was observed only between the RADm and RADdst. Conversely, the D_max_ to the optic chiasm was significantly higher for all RAD techniques than for RA (RADm, *p* = 0.008; RADa, *p* = 0.004; RADdst, *p* = 0.004). No significant differences were observed in the other dosimetric metrics.

For sinonasal cancer, significant overall differences were observed in seven OAR metrics, including the eyes, lenses, oral cavity, and parotid glands, using the Friedman test (*p* = 0.01 for the right eye and right parotid glands and < 0.01 for other OARs). For all the remaining OAR metrics, no significant differences were detected among the planning techniques. Pairwise comparisons revealed significant differences between the RA and RAD techniques, primarily for the eyes (RADm, *p* = 0.02; RADa, *p* = 0.01; RADdst, *p* = 0.01), lenses (RADm, *p* = 0.03; RADa, *p* = 0.01; RADdst, *p* = 0.01), oral cavity (RADm, *p* = 0.01; RADa, *p* = 0.01; RADdst, *p* = 0.01), and parotid glands (RADm, *p* = 0.02; RADa, *p* = 0.04; RADdst, *p* = 0.02).

### 3.3 Planning efficiency and delivery complexity

The results related to planning efficiency and delivery complexity are summarized in Table 1. The optimization calculation time was significantly reduced for all RAD techniques, with an average reduction of approximately 40% compared with RA (median: 242, 144, 143, and 140 s for RA, RADm, RADa, and RADdst, respectively, for nasopharyngeal cancer, *p* < 0.01; corresponding values of 241, 112, 104, and 106 s for sinonasal cancer, *p* < 0.01).

The estimated beam delivery time was also significantly shorter for the RAD plans, with an average reduction of approximately 20% relative to RA (median: 202, 172, 158, and 152 s in RA, RADm, RADa, and RADdst, respectively, for nasopharyngeal cancer, *p* < 0.01; corresponding values: 196, 145, 141, and 145 s for sinonasal cancer, *p* < 0.01). In addition, all RAD techniques exhibited a significantly higher MU and lower MCS than RA (*p* < 0.01), indicating a greater degree of beam modulation and delivery complexity.

## 4 DISCUSSION

In this study, we developed an ESAPI‐based decision support tool that automatically determines STAMP configurations for RAD planning by integrating BEV geometry with TrueBeam mechanical constraints. Although previous studies have reported the dosimetric advantages of RAD over conventional RA planning,^8^, ^9^, ^10^, ^11^, ^12^, ^13^ the automated determination of STAMP configurations has not been previously investigated. To the best of our knowledge, this is the first study to automate STAMP placement and evaluate its clinical utility in head and neck radiotherapy. The proposed workflow successfully generated dosimetrically feasible RAD plans for all the patients and significantly reduced the planning workload. The developed tool achieved a dosimetric performance that was comparable to both the time‐consuming manual STAMP planning and the vendor‐provided automated approach, thereby demonstrating that all RAD plans can be generated without sacrificing plan quality.

One of the most important findings of this study was that, although dose reductions among the RAD techniques were not observed across all OARs, the proposed geometric cost map framework effectively minimized the doses to several critical structures while maintaining an overall plan quality comparable to that of manual and commercial RAD approaches. Although the overall dosimetric profiles showed no clinically meaningful differences among RADm, RADa, and RADdst, this equivalence is highly encouraging from a clinical translation perspective. This demonstrates that the proposed automated tool can successfully replicate the plan quality of an experienced radiation oncologist and commercial algorithm, thereby supporting the validity of our underlying framework. The vendor‐provided RAD algorithm primarily determines collimator trajectories based on target‐shape characteristics to enhance the MLC modulation capability.^15^ In contrast, the proposed method explicitly incorporates both target conformity and OAR avoidance within the geometric cost map. Specifically, regarding the D_max_ to the optic chiasm and lens and D_mean_ to the oral cavity, the proposed RADdst tool reduced these OAR doses more effectively than RADm and RADa. This demonstrates the clear benefit of our geometric cost map optimization, which successfully enhances OAR‐avoidance capabilities by explicitly integrating critical structure locations into the trajectory generation process. Furthermore, these dosimetric advantages were achieved despite RADdst utilizing STAMP configurations that substantially differed from the standardized, expert‐defined gantry angles in RADm (Table S2). This spatial non‐uniqueness indicates that achieving high‐quality RAD plans does not rely on a single, rigid STAMP arrangement or human expert experience. Instead, the patient‐specific geometric optimization of RADdst successfully automates this decision‐making process, demonstrating that manual, trial‐and‐error STAMP configuration by experienced planners can be eliminated without sacrificing plan quality.

Another advantage of the proposed framework is its explainability. Unlike vendor‐provided automated algorithms, which provide limited insight into the rationale underlying the collimator trajectory selection, the proposed method determines STAMP locations using explicitly defined geometric cost functions and machine‐specific mechanical constraints. Consequently, each identified STAMP can be directly linked to a quantifiable geometric or mechanical requirement, allowing users to better understand the planning decisions made by the algorithm. Moreover, manually determining optimal STAMP configurations through visual inspection and trial and error is highly time‐consuming. In our study, during the initial development of the baseline RADm protocol for the first patient, the manual trial‐and‐error process, that is visually inspecting target/OAR geometry, testing candidate gantry/collimator angles, and evaluating iterative optimizations, was retrospectively estimated to require approximately 60 min of dedicated effort by the experienced radiation oncologist. In routine clinical practice, institutions often resort to re‐using standardized, fixed STAMP configurations across patients. Conversely, the proposed RADdst tool accomplished fully automated, patient‐customized STAMP determination in only 2–3 min without manual trial and error. Thus, rather than serving as a formal cohort‐wide timing benchmark, this comparison demonstrates that the tool effectively eliminates the initial manual setup workload while enabling individualized trajectory optimization for every patient.

The proposed framework offers a high degree of customization. Because the geometric cost function is defined explicitly, the weighting factors assigned to individual OARs can be modified to reflect specific clinical priorities. This enables physicians and planners to incorporate treatment‐specific objectives into the trajectory‐generation process before dose optimization is performed. This flexibility may facilitate broader clinical adoption across disease sites with different anatomical and dosimetric requirements.

In addition to maintaining dosimetric quality, the proposed workflow substantially improved overall planning efficiency by removing planner‐dependent tasks. The manual determination of STAMP configurations typically requires time‐consuming, trial‐and‐error adjustments. By automating this geometric decision‐making process, the proposed ESAPI tool successfully eliminated this manual workload, requiring only 2–3 min of user interaction. Regarding computational efficiency, all RAD techniques (RADm, RADa, and RADdst) reduced the optimization calculation time by approximately 40% relative to conventional RA planning. This calculation speedup across all RAD techniques is attributable to the reduced number of arcs (two coplanar arcs for RAD vs. three for RA) and the characteristics of the RAD optimization algorithm itself, rather than the specific STAMP configuration method. Furthermore, although all RAD techniques exhibited lower MCSs than RA, indicating increased modulation complexity, the estimated beam delivery time remained significantly shorter by approximately 20%. These findings suggest that increased plan modulation does not necessarily compromise delivery efficiency when collimator trajectories and STAMP locations are selected in a manner consistent with machine‐specific mechanical constraints.

This study has some limitations. First, this was a retrospective study with a relatively small sample size. Second, the evaluation was limited to nasopharyngeal and sinonasal cancers. Although the proposed geometric framework is not disease site‐specific, its performance should be validated at other anatomically complex treatment sites. Third, the weighting factors used in the geometric cost function were empirically selected. Future studies should investigate systematic approaches to optimize these parameters and evaluate the robustness of the framework across different clinical scenarios. Finally, this study was purely an in silico planning evaluation, without experimental phantom measurements, patient‐specific QA, trajectory log‐file analysis, or independent Monte Carlo calculations. Because RAD plans involve significantly increased modulation complexity (as reflected by higher MUs and lower MCSs) and dynamic collimator rotation, delivery accuracy and mechanical feasibility need to be rigorously verified on actual TrueBeam once clinical delivery capability becomes available at our institution.

## 5 CONCLUSION

We developed an ESAPI‐based automated decision support tool that determines cost‐optimized STAMP configurations for RAD planning by integrating BEV geometry with TrueBeam mechanical constraints. The proposed framework generated dosimetrically feasible RAD plans within hardware constraints, achieving dosimetric performance comparable to expert manual planning and vendor‐provided automatic optimization while eliminating manual, trial‐and‐error STAMP determination. Furthermore, the RAD approach demonstrated improved calculation efficiency and reduced estimated treatment delivery times compared with conventional RA. This method provides a practical pathway for automated and planner‐independent implementation of RAD in routine clinical practice.

## AUTHOR CONTRIBUTIONS

**Hideaki Hirashima**: Conceptualization; methodology; software; formal analysis; resources; data curation; investigation; writing—original draft; and funding acquisition. **Takahiro Iwai**: Formal analysis; resources; data curation; investigation; writing—review and editing; and funding acquisition. **Shinya Hiraoka**: Data curation; investigation; writing—review and editing; and funding acquisition. **Mitsuhiro Nakamura**: Data curation and writing—review and editing. **Ryota Nakashima**: Data curation and writing—review and editing. **Takashi Mizowaki**: Resources; supervision; project administration; writing—review and editing; and funding acquisition.

## CONFLICT OF INTEREST STATEMENT

Takahiro Iwai is employed through a commissioned research agreement with Varian Medical Systems. Hideaki Hirashima, Shinya Hiraoka, and Takashi Mizowaki are investigators in a commissioned research agreement supported by Varian Medical Systems. All other authors have no conflict of interest.

## ETHICAL APPROVAL

This retrospective study was approved by the Institutional Review Board of Kyoto University (approval number: 1446‐3).

## Supporting information




**Supporting Information**: acm270813‐sup‐0001‐Table S1.docx

**Supporting Information**: acm270813‐sup‐0002‐Table S2.docx

## Data Availability

The ESAPI script developed in this study is openly available in the GitHub repository at https://github.com/HideakiHirashima/ESAPI‐RAD‐STAMP‐Optimizer. The patient planning datasets generated and analyzed during the current study are available from the corresponding author upon reasonable request due to institutional privacy restrictions.
