# Supplementary material for: Automated decision support tool for static angle modulated ports configuration in VMAT with dynamic collimator rotation for head and neck cancer
Source: J Appl Clin Med Phys. 2026 Sep 23;27(10):e70813. doi: 10.1002/acm2.70813 (PMC13598925; doi:10.1002/acm2.70813)
Supplement: Supplementary file 1 [file ACM2-27-e70813-s001.docx]

**Supplementary Table 2**. Details of STAMPs in RADdst

|  |  | **Nasopharyngeal cancer** | | |  | **Sinonasal cancer** | | | | |
| --- | --- | --- | --- | --- | --- | --- | --- | --- | --- | --- |
| **No.** | **Arc** | **STAMPs (gantry angle/ collimator angle)** | | |  | **STAMPs (gantry angle/ collimator angle)** | | | | |
| 1 | Arc1  Arc2 | 310º/60º  0º/90º | 275º/60º  95º/120º | 180º/90º  130º/120º |  | 355º/180º  10º/90º | 305º/170º  80º/60º | 185º/40º  150º/60º |  |  |
| 2 | Arc1  Arc2 | 345º/100º  165º/80º |  |  |  | 335º/120º  0º/100º | 290º/130º  30º/60º | 245º/140º  65º/40º | 210º/120º  110º/50º | 180º/80º  155º/60º |
| 3 | Arc1  Arc2 | 275º/90º  0º/90º | 180º/90º  95º/90º |  |  | 335º/100º  65º/70º | 245º/100º  175º/80º |  |  |  |
| 4 | Arc1  Arc2 | 275º/90º  0º/90º | 180º/90º  95º/90º |  |  | 320º/140º  5º/70º | 255º/150º  50º/40º | 230º/140º  75º/30º | 185º/110º  140º/40º |  |
| 5 | Arc1  Arc2 | 345º/50º  10º/60º | 215º/90º  35º/90º | 190º/120º  165º/130º |  | 350º/100º  40º/90º | 245º/100º  65º/70º | 220º/90º  170º/70º |  |  |
| 6 | Arc1  Arc2 | 340º/70º  160º/110º |  |  |  | 345º/140º  5º/120º | 250º/130º  25º/100º | 205º/70º  70º/50º | 185º/60º  165º/40º |  |
| 7 | Arc1  Arc2 | 270º/90º  5º/60º | 185º/120º  90º/90º |  |  | 260º/130º  5º/80º | 185º/100º  80º/50º |  |  |  |
| 8 | Arc1  Arc2 | 335º/70º  0º/50º | 180º/130º  155º/110º |  |  | 275º/130º  0º/90º | 255º/130º  35º/80º | 215º/100º  75º/50º | 180º/90º  95º/50º |  |
| 9 | Arc1  Arc2 | 280º/50º  0º/80º | 180º/100º  100º/130º |  |  | 350º/130º  170º/50º |  |  |  |  |
| 10 | Arc1  Arc2 | 15º/90º  345º/90º |  |  |  | 285º/140º  0º/100º | 255º/140º  75º/40º | 180º/70º  105º/40º |  |  |

Abbreviation: STAMP, static angle-modulated port; RADdst, RAD with automated decision support tool.
