# Supplementary material for: Automated decision support tool for static angle modulated ports configuration in VMAT with dynamic collimator rotation for head and neck cancer
Source: J Appl Clin Med Phys. 2026 Sep 23;27(10):e70813. doi: 10.1002/acm2.70813 (PMC13598925; doi:10.1002/acm2.70813)
Supplement: Supplementary file 2 [file ACM2-27-e70813-s002.docx]

**Supplementary Table 1**. Dose constraints for nasopharyngeal and sinonasal cancers.

|  | Nasopharyngeal cancer | | | | Sinonasal cancer | | | |
| --- | --- | --- | --- | --- | --- | --- | --- | --- |
| Structure | Index | Dose constraints | | Priority | Index | Dose constraints | | Priority |
|  |  | Optimal | Acceptable |  |  | Optimal | Acceptable |  |
| PTV70 | D_95%_ | 70 Gy |  | Prescribed | D_50%_ | 70 Gy |  | Prescribed |
| PTV63 | D_50%_ | 63–64.58 Gy | 59.85–66.15 Gy | High | Same as nasopharyngeal cancer | | | |
| PTV56 | D_50%_ | 56–57.4 Gy | 53.2–58.8 Gy | High | - | - | - | - |
| GTV | D_98%_ | > 71.75 Gy | > 70 Gy | High | Same as nasopharyngeal cancer | | | |
| Spinal Cord | D_max_ | < 40 Gy | < 46 Gy | High | Same as nasopharyngeal cancer | | | |
| Brainstem | D_max_ | < 54 Gy | < 63 Gy | High | Same as nasopharyngeal cancer | | | |
| Optic nerve | D_max_ | < 50 Gy | < 55 Gy | High | Same as nasopharyngeal cancer | | | |
| Chiasm | D_max_ | < 50 Gy | < 55 Gy | High | Same as nasopharyngeal cancer | | | |
| CTV70 | D_98%_ | > 70 Gy | > 66.5 Gy | Low | D_95%_ | > 70 Gy | > 63 Gy | Low |
| CTV63 | D_95%_ | > 63 Gy | > 59.85 Gy | Low | Same as nasopharyngeal cancer | | | |
| CTV56 | D_95%_ | > 56 Gy | > 53.2 Gy | Low | - | - | - | - |
| Parotid (at least one) | D_mean_ | < 26 Gy | < 30 Gy | Low | Same as nasopharyngeal cancer | | | |
| Cochlea | D_mean_ | < 45 Gy | < 50 Gy | Low | Same as nasopharyngeal cancer | | | |
| Temporal lobe | D_max_ | < 63 Gy | < 74 Gy | Low | D_max_ | < 70 Gy | < 74 Gy | Low |
| Eyeball | D_max_ | < 35 Gy | < 45 Gy | Low | D_max_ (at least one) | < 45 Gy | < 50 Gy | Low |
| Lens | D_max_ | < 6 Gy | < 10 Gy | Low | Same as nasopharyngeal cancer | | | |
| Larynx | D_mean_ | < 30 Gy | < 45 Gy | Low | D_mean_ | < 45 Gy | < 50 Gy | Low |
| Oral cavity | D_mean_ | < 45 Gy | < 50 Gy | Low | Same as nasopharyngeal cancer | | | |
| Body | D_max_ | < 77 Gy | < 80.5 Gy | Low | Same as nasopharyngeal cancer | | | |

**Abbreviations**: CTV, clinical target volume; D_max_, maximum dose to the structure volume; D_mean_, mean dose to the structure volume; D_xx%_, maximum dose covering ≥ xx% of the structure volume; GTV, gross tumor volume; PTV, planning target volume.
